# Frontal Cerebral Blood Flow Deficits in Behavioral Variant Frontotemporal Dementia: Associations With Clinical Symptoms, Peripheral Vascular Biomarkers, and Neuroimaging Measures

**DOI:** 10.1002/cns.71235

**Published:** 2026-11-01

**Authors:** Jiahui Hou, Qianqian He, Xiuxia Fu, Yufei Chen, Hua Lu, Yuxuan Xu, Liyong Wu, Min Chu

**Affiliations:** ^1^ Department of Neurology, Xuanwu Hospital Capital Medical University Beijing China; ^2^ Yuhuangding Hospital Qingdao University Yantai Shandong China; ^3^ Qingdao Medical College Qingdao University Qingdao Shandong China; ^4^ National Clinical Research Center for Geriatric Diseases Beijing China

**Keywords:** arterial spin labeling, cerebral blood flow, frontotemporal dementia, mediation analysis, vascular biomarkers

## Abstract

**Aims:**

This study aimed to characterize regional cerebral blood flow (CBF) alterations in behavioral variant frontotemporal dementia (bvFTD), examine their associations with clinical symptoms and peripheral vascular/synaptic biomarkers, evaluate spatial concordance with gray matter volume (GMV) loss and glucose hypometabolism, and explore CBF's mediating role between biomarkers and clinical outcomes.

**Methods:**

Thirty‐five bvFTD patients and 27 matched normal controls (NC) underwent clinical assessments, ASL MRI, ^18^F‐FDG PET, and structural MRI. Plasma vascular (VEGFA, PDGF‐BB) and synaptic (NPTXR, NPTX2) biomarkers were measured via the Olink platform. Statistical analyses included group comparisons, ROC analysis, multiple linear regression, and mediation analysis.

**Results:**

Compared with normal controls, bvFTD patients exhibited significantly reduced CBF in both frontal and temporal regions, which showed regional spatial concordance with GMV and FDG uptake in corresponding areas. Frontal CBF demonstrated good diagnostic accuracy and was significantly associated with multiple clinical measures including MMSE, MoCA, ADL, and behavioral scales, as well as with peripheral vascular and synaptic biomarkers. Exploratory mediation analyses suggested statistical indirect associations linking peripheral vascular biomarkers to clinical symptoms through frontal CBF and linking frontal CBF to behavioral outcomes through frontal GMV.

**Conclusions:**

BvFTD is characterized by frontotemporal hypoperfusion, with frontal CBF closely associated with cognitive, behavioral, and functional impairment. The regional concordance of ASL‐derived CBF with gray matter atrophy and glucose hypometabolism supports its potential role as an accessible, non‐invasive, and radiation‐free complementary neuroimaging biomarker. The mediation findings should be considered hypothesis‐generating because they did not remain significant after FDR correction and require validation in larger longitudinal studies.

## Graphical abstract

Frontal and temporal hypoperfusion is observed in frontotemporal dementia, showing spatial concordance with gray matter atrophy and hypometabolism of glucose. Frontal hypoperfusion correlates significantly with peripheral vascular biomarkers and clinical phenotypes. Peripheral vascular factors affect clinical symptoms through CBF, while CBF affects clinical symptoms through GMV.

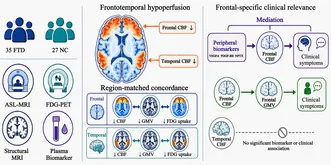

**Abbreviations**
3D‐pCASL: three‐dimensional pseudo‐continuous arterial spin labeling
ACC: anterior cingulate cortex
AD: Alzheimer's disease
ADL: activities of daily living
ASL: arterial spin labeling
BBB: blood‐brain barrier
bvFTD: behavioral variant frontotemporal dementia
CBF: cerebral blood flow
CDR: clinical dementia rating
ELISA: enzyme‐linked immunosorbent assay
FBI: frontal Behavioral Inventory
FDG‐PET: ^18^F‐fluorodeoxyglucose positron emission tomography
FTD: frontotemporal dementia
FTLD: frontotemporal lobar degeneration
FTLD‐CDR: frontotemporal lobar degeneration clinical dementia rating
GM: gray matter
GMV: gray matter volume
MMSE: mini‐mental state examination
MoCA: montreal cognitive assessment
MRI: magnetic resonance imaging
NC: normal controls
nfvPPA: non‐fluent variant primary progressive aphasia
NPI‐Q: neuropsychiatric inventory questionnaire
NPTX2: neuronal pentraxin 2
NPTXR: neuronal pentraxin receptor
pCASL: pseudo‐continuous arterial spin labeling
PDGF‐BB: platelet‐derived growth factor BB
PET: positron emission tomography
PFC: prefrontal cortex
ROC: receiver operating characteristic
svPPA: semantic variant primary progressive aphasia
TDP‐43: tar DNA‐binding protein 43
VEGFA: vascular endothelial growth factor A

## 1 Introduction

Frontotemporal dementia (FTD) is the second most common cause of early‐onset dementia after Alzheimer's disease and typically manifests before the age of 65 years [1]. It encompasses several clinically distinct syndromes, principally behavioral variant frontotemporal dementia (bvFTD), semantic variant primary progressive aphasia, and nonfluent/agrammatic variant primary progressive aphasia [2]. Among these syndromes, bvFTD is the most common subtype and is characterized by progressive changes in behavior, personality, and executive function [3]. Despite advances in understanding the molecular pathology of bvFTD, reliable and accessible biomarkers for early diagnosis and disease monitoring remain an unmet clinical need.

Neuroimaging has been instrumental in characterizing the structural and functional signatures of bvFTD. Structural MRI consistently demonstrates frontal and anterior temporal atrophy, although significant cortical atrophy typically becomes apparent only at relatively later stages of bvFTD [4]. Glucose hypometabolism in the prefrontal cortex (PFC), anterior cingulate cortex (ACC), and anterior temporal cortex may be observed as early as the initial stages on ^18^F‐fluorodeoxyglucose positron emission tomography (^18^F‐FDG PET) scans [5, 6]. Although FDG‐PET provides valuable metabolic information, its clinical utility is limited by high cost, restricted availability, exposure to ionizing radiation, and the requirement for radioactive tracers [7]. Consequently, there is growing interest in developing accessible, non‐invasive neuroimaging approaches that may provide complementary information about related pathophysiological changes.

Arterial spin labeling (ASL) MRI offers non‐invasive quantification of cerebral blood flow (CBF) without the need for exogenous contrast agents or radioactive tracers, making it a promising candidate for clinical application [8, 9, 10, 11]. CBF is tightly coupled to neuronal activity and cerebral metabolism through neurovascular mechanisms, and chronic hypoperfusion may contribute to neurodegeneration via multiple pathways, including impaired clearance of pathological proteins, energy deprivation, oxidative stress, and blood–brain barrier (BBB) disruption [12, 13]. Consistent with this, prior studies have demonstrated that bvFTD is associated with concurrent prefrontal hypometabolism and reduced cerebral perfusion [9], and ASL‐derived CBF patterns in neurodegenerative diseases have been shown to closely mirror FDG‐PET hypometabolism, owing to the tight coupling between neuronal activity, glucose consumption, and regional blood flow. Beyond characterizing disease‐specific perfusion signatures, ASL has also demonstrated utility in differentiating FTD from AD: posterior cingulate perfusion is characteristically lower in AD than in FTD, reflecting the distinct topographic vulnerabilities of each condition [14]. Nevertheless, the application of ASL in bvFTD remains comparatively limited, and the mechanisms underlying CBF reductions in bvFTD, as well as their relationships with clinical phenotypes, have yet to be fully elucidated.

Building on this evidence, the present study used ASL MRI to: (1) characterize CBF reductions in the frontal and temporal regions of patients with bvFTD; (2) examine the associations of frontal and temporal CBF with clinical measures; (3) assess the regional associations of CBF with gray Matter Volume (GMV) and FDG uptake; (4) investigate the associations of regional CBF with peripheral vascular and synaptic biomarkers; and (5) use exploratory mediation analyses to evaluate potential statistical indirect associations among peripheral biomarkers, regional CBF, GMV, FDG uptake, and clinical outcomes.

## 2 Methods and Materials

### 2.1 Ethics

This study was conducted following the Declaration of Helsinki. The clinical protocols were approved by the ethics committee and local institutional review board of Xuanwu Hospital, Capital Medical University, China (approval number: 2020026). The study was conducted following relevant guidelines and regulations for the use of human subjects in research. Written informed consent was obtained from all participants or their guardians before the start of the study.

### 2.2 Participants

This cross‐sectional study enrolled 35 patients with probable bvFTD from the Department of Neurology, Xuanwu Hospital, Capital Medical University, and 27 cognitively normal controls (NC) recruited from the community. Controls were matched to the bvFTD group for age, sex, and years of education. All patients met the 2011 consensus criteria for probable bvFTD [15], and the diagnoses were confirmed by a multidisciplinary team of experienced neurologists, neuropsychologists, and neuroradiologists. Additional inclusion criteria for patients were the availability of complete clinical assessment data and the ability to undergo multimodal neuroimaging. Exclusion criteria included serious neurological or psychiatric disorders that could affect cognition, including substance abuse, alcoholism, schizophrenia, brain tumors, or cerebrovascular disease, as well as contraindications to MRI. Patients without a reliable informant were also excluded. NC participants had no clinical evidence of cognitive impairment, depression, anxiety, or other neurological or psychiatric disorders. They also met the predefined cognitive screening criteria, including an MMSE score ≥ 26 and scores of 0 in both the behavior/comportment and language domains of the CDR plus NACC FTLD module.

### 2.3 Clinical Assessments

All participants underwent a standardized battery of clinical evaluations administered by trained neuropsychologists blinded to neuroimaging results. Global cognitive function was assessed using the MMSE and MoCA. Disease severity was evaluated using the FTLD‐modified CDR (FTLD‐CDR), which includes two additional domains (behavior and language) compared to the standard CDR, yielding an FTLD‐CDR sum of boxes score [16]. The FTLD‐CDR subscales assessed memory, orientation, judgment and problem solving, social affairs, hobbies, self‐care, behavior, and language. Functional status was evaluated using the ADL scale. Behavioral symptoms were quantified using the NPI‐Q and the FBI, which provide separate apathy and disinhibition subscale scores as well as a total score.

### 2.4 Plasma Biomarker Measurements

Venous blood samples were collected in EDTA‐coated tubes, centrifuged at 2000 ×*g* for 15 min at 4°C within 2 h, and stored at −80°C. One microliter of plasma was analyzed using the Olink Inflammation Reagent Kit (no. 95302) based on proximity extension assay technology. The assays were performed by Sinotech Genomics Co. Ltd. (Shanghai, China), using an Olink Signature Q100 instrument. VEGFA, PDGF‐BB, NPTXR, and NPTX2 were reported as normalized protein expression values. NPX is an arbitrary unit on a log_2_ scale representing relative protein abundance.

### 2.5 Neuroimaging Analysis

#### 2.5.1 ^18^ F‐FDG PET/MRI Acquisition Parameters

All images were acquired on a hybrid 3.0 T time‐of‐flight ^18^F‐fluorodeoxyglucose (FDG)–PET/MRI scanner (SIGNA FDG–PET/MR, GE Healthcare, WI, USA). FDG–PET and MRI data were acquired simultaneously using a vendor‐supplied 19‐channel head and neck union coil. Three‐dimensional (3D) T1‐weighted sagittal images and FDG–PET volumes were acquired during the same session after administering 3.7 MBq/kg ^18^F‐FDG.

The parameters of the T1 data were as follows: repetition time (TR) = 6.9 ms, echo time (TE) = 2.98 ms, flip angle = 12°, inversion time = 450 ms, matrix size = 256 × 256, field of view (FOV) = 256 × 256mm^2^, slice thickness = 1 mm, 192 sagittal slices with no gap, voxel size = 1 × 1 × 1mm^3^, and acquisition time = 4 min 48 s. Static ^18^F‐FDG–PET data were acquired using the following parameters: matrix size = 192 × 192, FOV = 350 × 350 mm^2^, and pixel size = 1.82 × 1.82 × 2.78 mm^3^, including corrections for random coincidences, dead time, scatter, and photon attenuation. Attenuation correction was performed based on MRI of the brain (Atlas‐based coregistration of two‐point Dixon). The default attenuation correction sequence was automatically prescribed and acquired as follows: LAVA‐Flex (GE Healthcare) axial acquisition, TR = 4 ms, TE = 1.7 ms, slice thickness = 5.2 mm with a 2.6 mm overlap, 120 slices, pixel size = 1.95 × 2.93 mm, and acquisition time = 18 s.

Pulsed arterial spin labeling (PASL) three‐dimensional sequence was adopted, with a labeling duration of 2 s, TR = 4000 ms, TE = 19.82 ms, flip angle of 180°, matrix size = 64 × 64, FOV = 192 × 192 mm^2^, slice thickness = 3 mm, 48 axial slices with no gap, voxel size = 3 × 3 × 3 mm^3^, number of excitations (NEX) = 3, and a total scan time of approximately 6 min and 28 s.

#### 2.5.2 Neuroimaging Preprocessing

Data were preprocessed using the computational anatomy toolbox 12 (CAT12) (http://www.neuro.uni‐jena.de/cat/), which is based on statistical parametric mapping12 (SPM12). First, the DICOM files were converted into NIfTI format using MRIcron software (http://people.cas.sc.edu/rorden/mricron/index.html). Voxel‐based morphometry preprocessing was performed using the default settings of the CAT12 toolbox and the “East Asian Brains” ICBM template. T1‐weighted 3D images were segmented into gray matter (GM), white matter, and cerebrospinal fluid partitions. Subsequently, the GM and WM partitions of each subject in native space were high dimensionally registered and normalized to the standard Montreal Neurological Institute space using diffeomorphic anatomical registration through exponentiated lie algebra normalization. The images were then smoothed using an 8 mm full‐width half‐maximum Gaussian kernel.

#### 2.5.3 FDG PET Image Preprocessing

FDG–PET images were preprocessed using SPM12, implemented in MATLAB (MathWorks, Natick, Massachusetts). After normalization of the structural MRI images, the transformation parameters determined by the T1‐weighted image spatial normalization were applied to the coregistered FDG–PET images for FDG–PET spatial normalization. The images were then smoothed using an isotropic Gaussian kernel with an 8 mm full‐width half‐maximum. Finally, FDG–PET scan intensity was normalized using a whole cerebellum reference region to create standardized uptake value ratio images.

#### 2.5.4 Regions of Interest (ROI) Analysis

We performed an atlas‐based ROI analysis of structural MRI, ASL, and FDG‐PET images, selecting the frontotemporal lobe as the brain region of interest for further linear regression correlation analysis.

### 2.6 Statistical Analysis

Statistical analyses were performed using R (version 4.3.0; R Foundation for Statistical Computing, Vienna, Austria). Continuous variables were assessed for normality using the Shapiro–Wilk test. Demographic and clinical characteristics between bvFTD and NC groups were compared using Mann–Whitney *U* tests for continuous variables and chi‐square tests for categorical variables. Data are presented as median [interquartile range] for continuous variables and *n* (%) for categorical variables.

Receiver operating characteristic (ROC) curve analysis was performed to evaluate the discriminative capacity of frontotemporal CBF for differentiating bvFTD from NC. The area under the curve (AUC), sensitivity, specificity, and optimal cutoff values were determined using the Youden index method.

Before regression analysis, data completeness, variable distributions, and potential outliers were examined. Continuous variables were standardized as *z*‐scores. Separate multiple linear regression models assessed the associations of frontal or temporal CBF with clinical, neuroimaging, and plasma biomarker measures. Age, sex, and education were included as covariates in all regression and mediation models. Residual normality, homoscedasticity, linearity, multicollinearity, and influential observations were assessed using the Shapiro–Wilk test, Breusch–Pagan test, Ramsey RESET test, variance inflation factors, and Cook's distance, respectively. Standardized regression coefficients (*β*), two‐sided *p* values, and Benjamini–Hochberg false discovery rate (FDR)‐adjusted *p* values were reported.

Exploratory mediation analyses evaluated two groups of candidate pathways: (1) peripheral vascular biomarkers–regional CBF–clinical outcomes and (2) regional CBF–regional GMV or FDG uptake–clinical outcomes. Linear regression models were fitted based on the method proposed by Baron and Kenny [17]. Reverse mediation analyses were conducted as sensitivity analyses by exchanging the independent and outcome variables while retaining the mediator specified in the primary model.

## 3 Results

### 3.1 Participant Characteristics

As shown in Table 1, there were no significant differences between bvFTD and NC groups in demographic characteristics (sex, age, education, all *p* > 0.05). bvFTD patients demonstrated significantly worse cognitive function, greater disease severity, impaired daily living ability, and more severe behavioral symptoms compared with NC (all *p* < 0.001). All FTLD‐CDR domain subscale scores were significantly higher in bvFTD patients (all *p* < 0.001).

**TABLE 1:** Demographic and clinical characteristics of study participants.

Characteristics	bvFTD	NC	*p*
*N*	35	27	
Female (%)	22 (62.9)	16 (59.3)	0.798
Age	63.00 [59.50, 67.00]	61.00 [60.00, 65.50]	0.522
Education	9.00 [7.00, 13.50]	9.00 [9.00, 10.50]	0.777
Global cognition			
MMSE	15.00 [9.50, 19.50]	29.00 [28.00, 29.50]	**< 0.001**
MoCA	10.00 [5.50, 13.00]	26.00 [25.00, 27.00]	**< 0.001**
FTLD‐CDR	10.00 [7.00, 13.50]	0.00 [0.00, 0.00]	**< 0.001**
Cognitive domain			
Memory	1.00 [1.00, 2.00]	0.00 [0.00, 0.00]	**< 0.001**
Orientation	1.00 [0.50, 1.50]	0.00 [0.00, 0.00]	**< 0.001**
Judgment	1.00 [0.50, 2.00]	0.00 [0.00, 0.00]	**< 0.001**
SocialAffair	1.00 [0.50, 2.00]	0.00 [0.00, 0.00]	**< 0.001**
Hobby	1.00 [0.50, 2.00]	0.00 [0.00, 0.00]	**< 0.001**
Self‐care	1.00 [0.25, 1.00]	0.00 [0.00, 0.00]	**< 0.001**
Behavior	2.00 [2.00, 3.00]	0.00 [0.00, 0.00]	**< 0.001**
Language	1.00 [0.25, 2.00]	0.00 [0.00, 0.00]	**< 0.001**
Activities of daily living			
ADL	29.00 [22.50, 42.50]	20.00 [20.00, 20.00]	**< 0.001**
Behavioral symptoms			
NPI‐Q	24.00 [13.00, 31.50]	0.00 [0.00, 0.00]	**< 0.001**
FBI‐apathy	18.00 [12.00, 22.00]	0.00 [0.00, 0.00]	**< 0.001**
FBI‐disinhibition	6.00 [2.00, 8.50]	0.00 [0.00, 0.00]	**< 0.001**
FBI total	21.00 [14.00, 32.50]	0.00 [0.00, 0.00]	**< 0.001**

*Note:* Bold *p* values indicate statistical significance (*p* < 0.05).

Abbreviations: ADL, activities of daily living; bvFTD, behavioral variant frontotemporal dementia; FBI, frontal behavioral inventory; FTLD‐CDR, frontotemporal lobar degeneration clinical dementia rating; MMSE, mini‐mental state examination; MoCA, montreal cognitive assessment; NC, normal controls; NPI‐Q, neuropsychiatric inventory questionnaire.

### 3.2 Frontotemporal Hypoperfusion in bvFTD

As is shown in Figure 1, compared with NC, bvFTD patients demonstrated significantly reduced CBF in both frontal and temporal cortical regions (*p* < 0.001).

**FIGURE 1:**
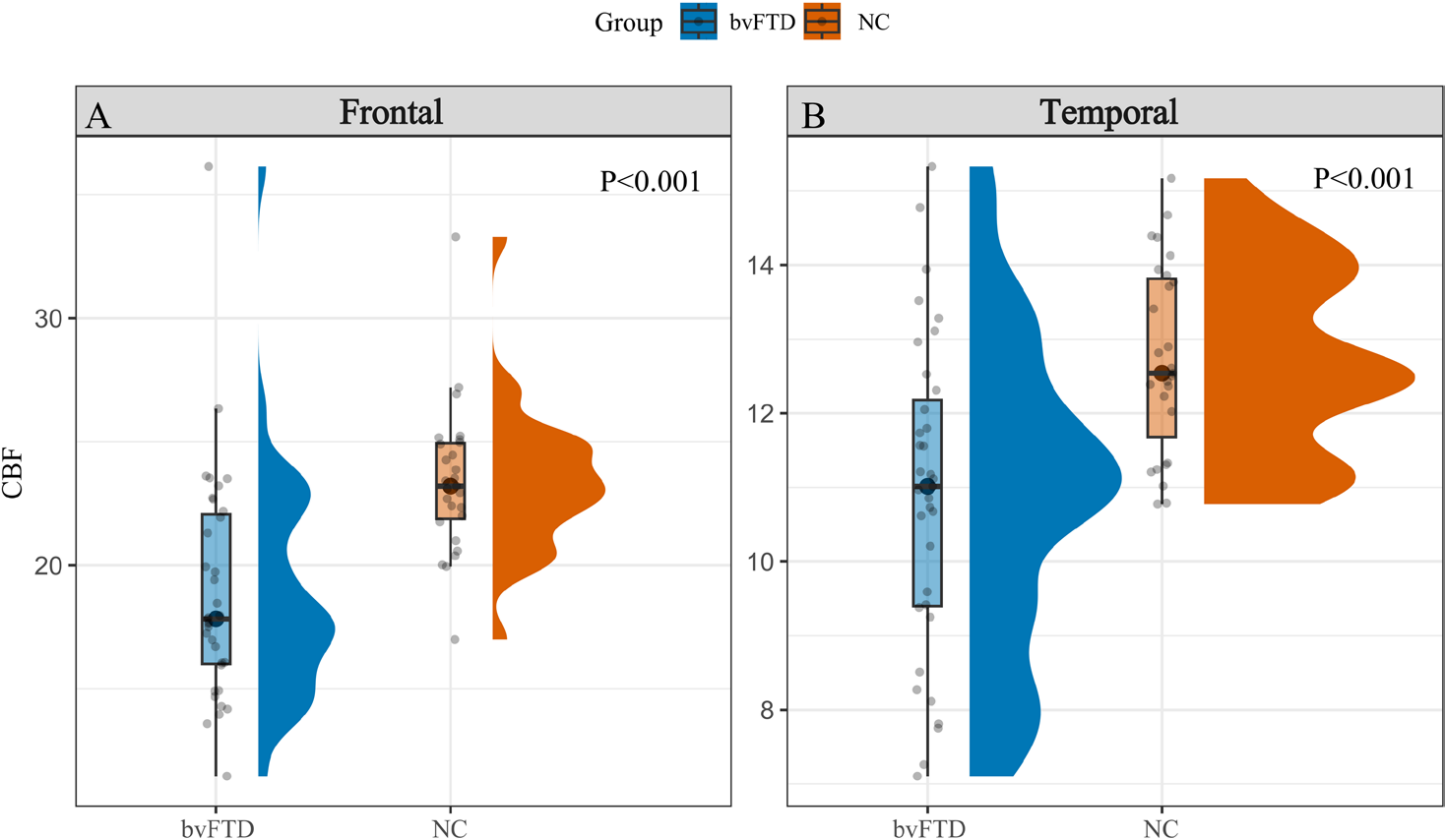
The intergroup differences in regional cerebral blood flow between bvFTD patients and NC. (A) Frontal CBF. (B) Temporal CBF. bvFTD, behavioral variant frontotemporal dementia; NC, normal controls.

### 3.3 Discriminative Accuracy of Frontotemporal CBF

ROC curve analysis demonstrated that frontotemporal CBF values provided good discriminative accuracy for differentiating bvFTD patients from NC (File S1). The AUC for frontal CBF was 0.825, and the AUC for temporal CBF was 0.769.

### 3.4 Associations Between Frontal CBF and Clinical Symptoms

Multiple linear regression analyses showed significant associations between frontal CBF and several clinical measures after FDR correction (Figure 2 and File S2). Higher frontal CBF was associated with better global cognitive performance, as reflected by higher MMSE (*β* = 0.794, FDR‐*p* < 0.001) and MoCA scores (*β* = 0.790, FDR‐*p* < 0.001), and with lower disease severity and functional impairment, as indicated by lower FTLD‐CDR (*β* = −0.883, FDR‐*p* < 0.001) and ADL scores (*β* = −0.609, FDR‐*p* = 0.001). Among the FTLD‐CDR domains, higher frontal CBF was associated with lower scores for memory (*β* = −0.743, FDR‐*p* < 0.001), orientation (*β* = −0.665, FDR‐*p* = 0.001), judgment (*β* = −0.757, FDR‐*p* < 0.001), social affairs (*β* = −0.707, FDR‐*p* < 0.001), hobbies (*β* = −0.745, FDR‐*p* < 0.001), and self‐care (*β* = −0.476, FDR‐*p* = 0.019). Frontal CBF was also negatively associated with FBI‐disinhibition (*β* = −0.519, FDR‐*p* = 0.008) and FBI‐total scores (*β* = −0.488, FDR‐*p* = 0.018). Associations with behavior, language, NPI‐Q, and FBI‐apathy did not remain significant after FDR correction (all FDR‐*p* ≥ 0.052). Temporal CBF was not significantly associated with any clinical measure after FDR correction (all FDR‐*p* ≥ 0.311).

**FIGURE 2:**
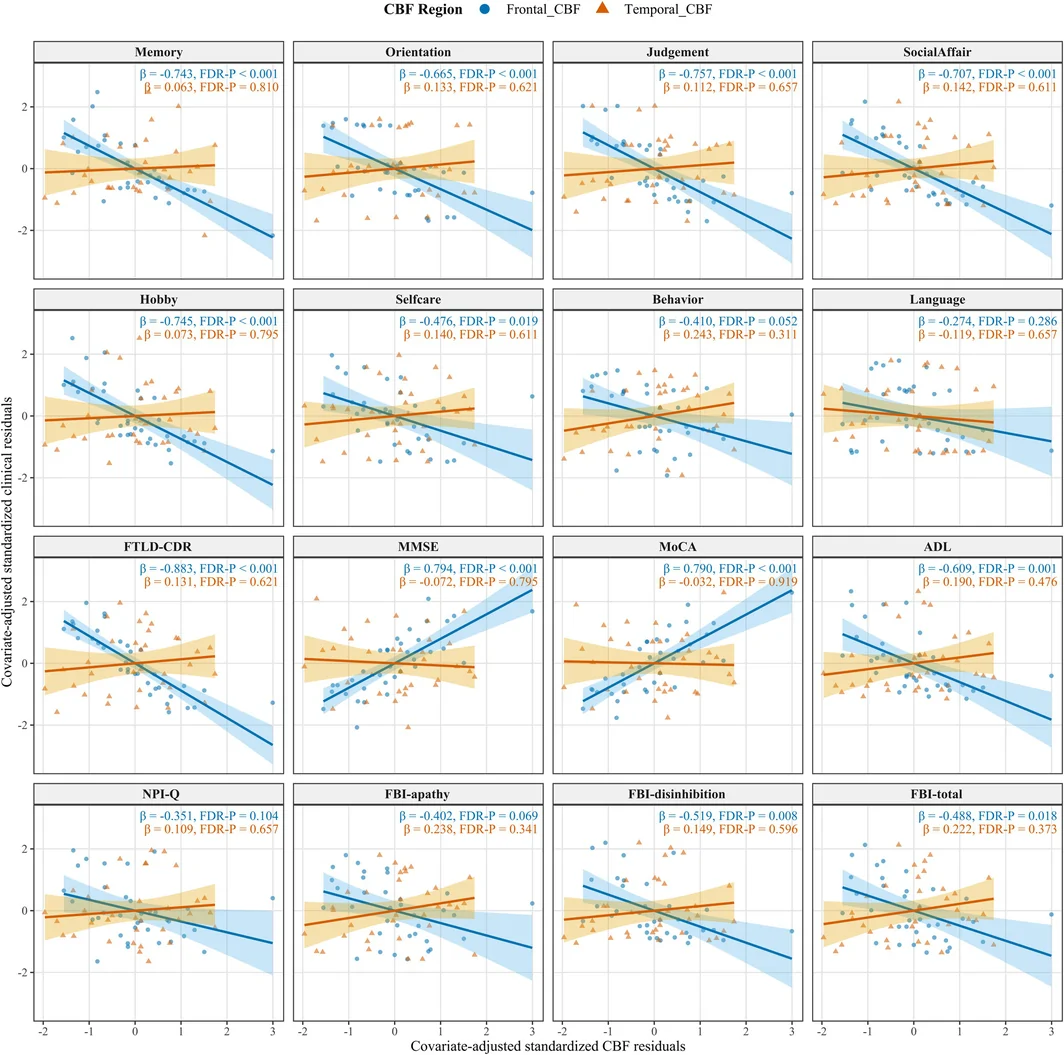
Linear correlation between Frontotemporal CBF and clinical symptoms in bvFTD patients. ADL, activities of daily living; bvFTD, behavioral variant frontotemporal dementia; FBI, frontal behavioral inventory; FTLD‐CDR, frontotemporal lobar degeneration clinical dementia rating; MMSE, mini‐mental state examination; MoCA, montreal cognitive assessment; NC, normal controls; NPI‐Q, neuropsychiatric inventory questionnaire.

### 3.5 Regional Associations of CBF With GMV and FDG Uptake

The associations of regional CBF with GMV and FDG uptake are presented in Table 2. After FDR correction, frontal CBF was positively associated with frontal GMV (β = 0.573, FDR‐*p* = 0.006) and frontal FDG uptake (*β* = 0.519, FDR‐*p* = 0.008), whereas its associations with temporal GMV and temporal FDG uptake were not significant (FDR‐*p* = 0.596 and 0.989, respectively). Temporal CBF was positively associated with temporal GMV (*β* = 0.539, FDR‐*p* = 0.006), but its association with temporal FDG uptake did not remain significant after FDR correction (*β* = 0.334, FDR‐*p* = 0.071). Temporal CBF was also not significantly associated with frontal GMV or frontal FDG uptake (FDR‐*p* = 0.591 and 0.283, respectively). These findings support regionally corresponding associations between CBF and GMV in both frontal and temporal regions, whereas a significant regional association between CBF and glucose metabolism was observed only in the frontal region.

**TABLE 2:** Associations between regional CBF and neuroimaging measures in bvFTD patients.

CBF	Neuroimaging measure	*β*	*p*	FDR‐P
Frontal_CBF	Frontal_GMV	0.573	**0.001**	**0.006**
	Temporal_GMV	0.161	0.394	0.596
	Frontal_FDG	0.519	**0.002**	**0.008**
	Temporal_FDG	0.007	0.968	0.989
Temporal_CBF	Frontal_GMV	−0.166	0.369	0.591
	Temporal_GMV	0.539	**0.001**	**0.006**
	Frontal_FDG	−0.257	0.142	0.283
	Temporal_FDG	0.334	**0.031**	0.071

*Note:* Bold values in the *p* column indicate unadjusted *p* < 0.05; bold values in the FDR‐P column indicate FDR‐adjusted *p* < 0.05.

Abbreviations: CBF, cerebral blood flow; FDG, ^18^F‐fluorodeoxyglucose uptake; GMV, gray matter volume; FDR, false discovery rate.

### 3.6 Associations Between Frontal CBF and Peripheral Biomarkers

The associations between regional CBF and plasma biomarkers are summarized in Figure 3 and File S3. After FDR correction, frontal CBF was negatively associated with plasma NPTX2 (*β* = −0.415, FDR‐*p* = 0.043), VEGFA (*β* = −0.456, FDR‐*p* = 0.033), and PDGF‐BB (*β* = −0.558, FDR‐*p* = 0.006). The association between frontal CBF and NPTXR did not remain significant after FDR correction (*β* = −0.345, FDR‐*p* = 0.100). Temporal CBF was not significantly associated with any of the examined biomarkers after FDR correction (all FDR‐*p* ≥ 0.621).

**FIGURE 3:**
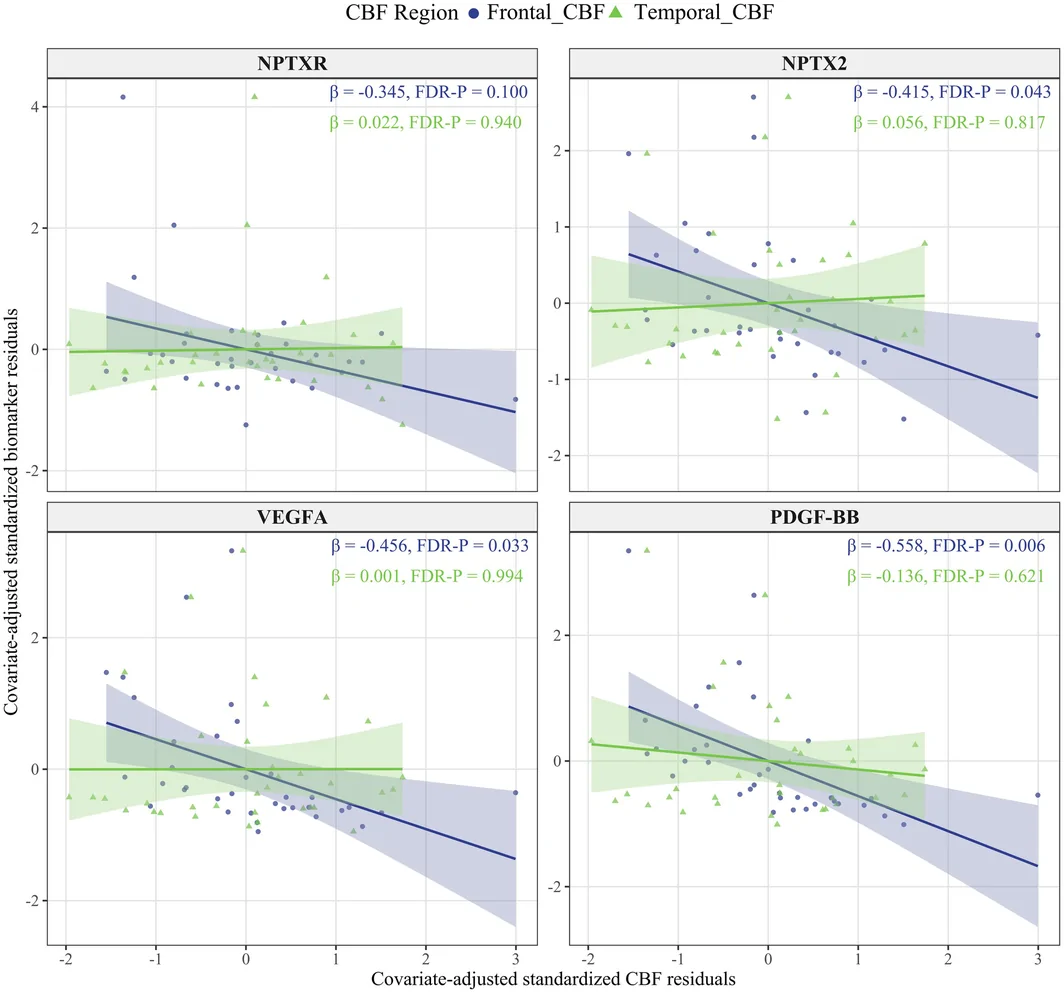
Linear correlation between frontal CBF and peripheral biomarker in bvFTD patients. bvFTD, behavioral variant frontotemporal dementia; NC, normal controls; NPTXR, neuronal pentraxin receptor; PDGF‐BB, platelet‐derived growth factor BB; NPTX2, neuronal pentraxin 2; VEGFA, vascular endothelial growth factor A.

### 3.7 Mediation Analysis

Exploratory mediation analyses identified several significant indirect associations in the primary models (*p* < 0.05), although none remained significant after FDR correction. As shown in Figure 4A–F and File S4, frontal CBF showed significant indirect associations linking VEGFA and PDGF‐BB with FTLD‐CDR (proportion mediated: 83%–88%), MMSE (68%–74%), judgment (62%–79%), ADL (52%–74%), self‐care (41%–59%), and orientation (68%). These findings suggest that frontal CBF may statistically account for part of the observed associations between peripheral vascular biomarkers and clinical outcomes.

**FIGURE 4:**
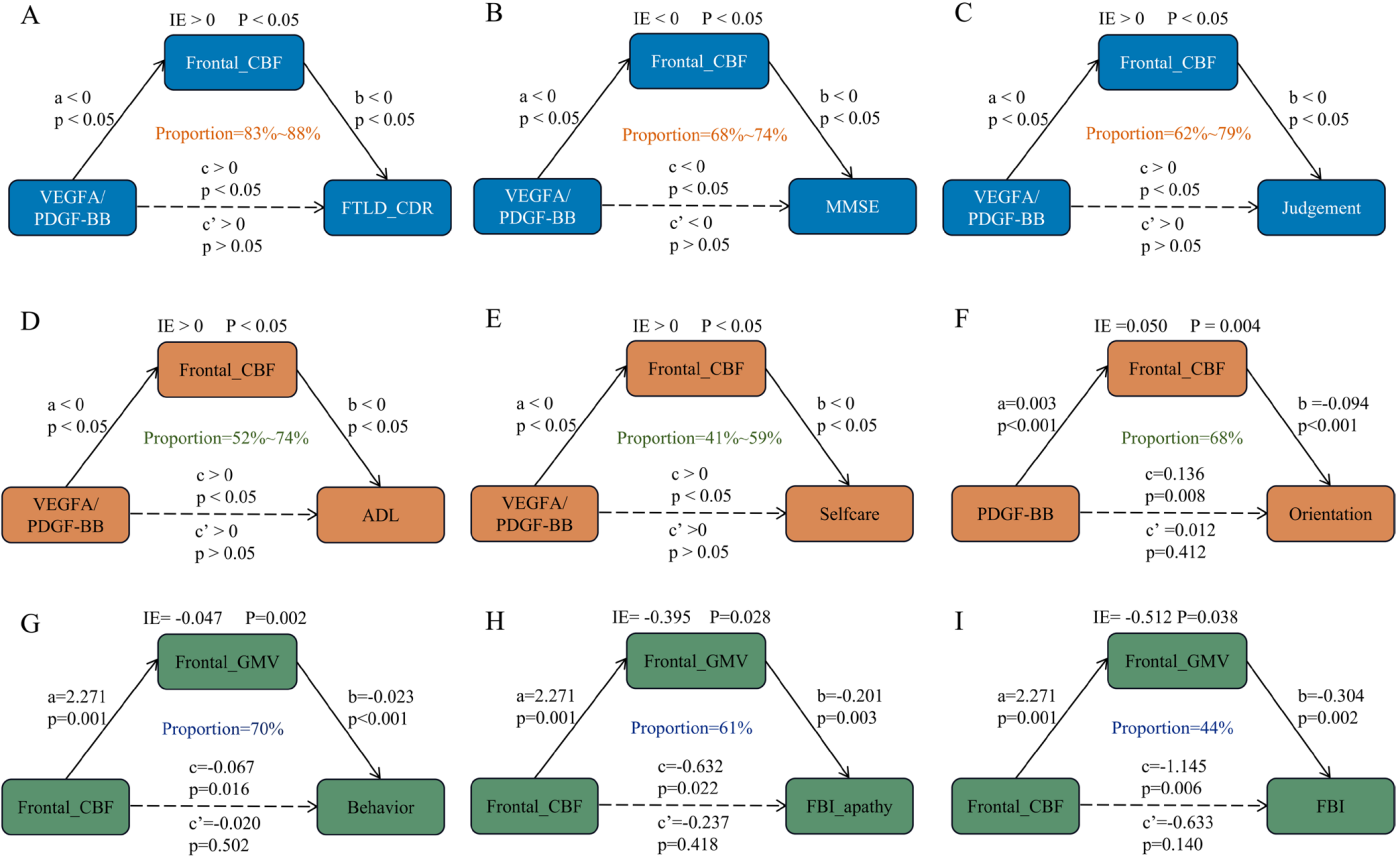
Exploratory mediation analyses. (A‐F) Frontal CBF as the mediator between VEGFA/PDGF‐BB and (A) FTLD‐CDR, (B) MMSE, (C) judgment, (D) ADL,(E) self‐care, and (F) orientation. (G‐I) Frontal GMV as the mediator between frontal CBF and (G) behavior, (H) FBI‐apathy, and (I) FBI total score. a is the effect of the independent variable on mediators; b is the effect of mediators on dependent variables after controlling the influence of independent variables; c is the total effect of independent variables on dependent variables; c’ is the direct effect of independent variables on dependent variables after controlling the influence of mediators; IE is the indirect effect of independent variables on dependent variables, in this intermediary model, c = c’ + ab.

As shown in Figure 4G–I and File S5, frontal GMV showed significant indirect associations linking frontal CBF with behavior (proportion mediated: 70%), FBI apathy (61%), and FBI total scores (44%). In the reverse mediation analyses, six indirect associations were significant (*p* < 0.05; File S6), but none remained significant after FDR correction. Therefore, all mediation findings should be considered exploratory.

## 4 Discussion

This study provides a multimodal characterization of CBF alterations in bvFTD and their associations with clinical manifestations, peripheral biomarkers, and other neuroimaging measures. Patients with bvFTD exhibited prominent frontal and temporal hypoperfusion, with frontal CBF showing significant associations with cognitive, behavioral, and functional impairment after FDR correction. Frontal CBF was associated with frontal GMV and FDG uptake, whereas temporal CBF was associated with temporal GMV but not temporal FDG uptake after FDR correction. Frontal CBF was also inversely associated with selected peripheral vascular and synaptic biomarkers, including VEGFA, PDGF‐BB, and NPTX2. Exploratory mediation analyses identified indirect associations linking peripheral vascular biomarkers to clinical outcomes via frontal CBF and linking frontal CBF to behavioral outcomes via frontal GMV.

### 4.1 Frontotemporal Hypoperfusion in bvFTD

The reductions in frontal and temporal CBF observed in patients with bvFTD are consistent with the characteristic distribution of neurodegeneration in this disorder and with previous ASL studies [9, 14]. This regional pattern also broadly parallels the distributions of gray matter atrophy and glucose hypometabolism reported using structural MRI and FDG‐PET [14, 18]. Whereas previous studies have primarily described group‐level perfusion abnormalities or evaluated the diagnostic performance of ASL, the present study integrated ASL, structural MRI, FDG‐PET, detailed clinical assessments, and peripheral vascular and synaptic biomarkers within the same cohort. This multimodal approach enabled direct examination of the regional associations of CBF with structural, metabolic, clinical, and peripheral biomarker measures.

### 4.2 Frontal CBF as a Clinically Meaningful Biomarker

A key finding of this study is the different clinical associations of frontal and temporal CBF. Frontal CBF was significantly associated with global cognition, disease severity, daily functioning, and behavioral symptoms, whereas temporal CBF showed no significant associations with the clinical measures examined. This pattern may partly reflect the characteristics of our cohort, which consisted exclusively of patients with bvFTD, a syndrome in which frontal behavioral‐executive network dysfunction plays a predominant role in the clinical phenotype [19, 20, 21]. In addition, our clinical battery focused mainly on global cognition, behavioral symptoms, disease severity, and daily functioning and may therefore have been more sensitive to frontal dysfunction. Although the FTLD‐CDR includes a language domain, it provides a relatively broad clinical rating and may not fully capture specific temporal‐lobe functions, such as semantic processing, emotion recognition, and person‐specific knowledge [22]. Importantly, temporal CBF was significantly reduced in patients with bvFTD, indicating temporal‐lobe involvement. Therefore, the absence of significant temporal CBF‐clinical associations should not be interpreted as evidence that temporal hypoperfusion is clinically irrelevant.

### 4.3 Spatial Concordance Between CBF, Atrophy, and Metabolism

The regional double dissociation observed between CBF, GMV, and FDG uptake—where frontal CBF correlated selectively with frontal but not temporal GMV and FDG, and vice versa—provides strong evidence for spatial concordance across these neuroimaging modalities. This finding extends previous observations in AD to the FTD spectrum and has important clinical implications [23, 24]. Given the cost, limited availability, and radiation exposure associated with FDG‐PET, our findings support the potential role of ASL‐derived CBF as an accessible, non‐invasive, and radiation‐free complementary approach for assessing regional brain dysfunction in bvFTD. The concordance likely reflects the tight coupling between neuronal activity, glucose metabolism, and cerebral blood flow, which is maintained even in the context of neurodegeneration [25]. Future studies directly comparing the diagnostic and prognostic performance of ASL and FDG‐PET in larger, multi‐center cohorts will be needed to formally establish the clinical equivalence of these modalities.

### 4.4 Peripheral Vascular and Synaptic Biomarkers in Relation to CBF

To our knowledge, this is the first study to examine the relationships between peripheral vascular/synaptic biomarkers and cerebral perfusion in bvFTD. VEGFA is induced by hypoxia and may promote angiogenesis [26], whereas PDGF‐BB/PDGFRβ signaling supports pericyte survival and vascular integrity [27]. Thus, elevated plasma VEGFA and PDGF‐BB may represent compensatory responses to cerebral hypoperfusion or vascular injury. Alternatively, these elevations may reflect systemic endothelial activation, platelet‐related release, inflammation, or dysregulated vascular signaling. Excessive VEGFA signaling may also increase BBB permeability and impair capillary flow [28, 29], indicating that elevated vascular growth factors are not necessarily protective.

NPTX2 and NPTXR are involved in synaptic organization and plasticity [30, 31]. The inverse association between plasma NPTX2 and frontal CBF cannot be explained by reduced synaptic activity alone and may reflect neurodegeneration‐related release, altered BBB transport, peripheral clearance, or systemic sources. Reports of reduced neuronal pentraxins in CSF but elevated plasma NPTX2 in genetic FTD support potential compartment‐specific differences [32, 33]. Circulating neuronal pentraxins should therefore not be interpreted as direct measures of their expression or activity in the brain. Longitudinal studies with paired plasma and CSF measurements are needed to clarify these associations.

### 4.5 Exploratory Mediation Findings

The exploratory mediation analyses suggested two potential patterns of statistical indirect association. First, nominally significant indirect associations were observed between peripheral vascular biomarkers and clinical outcomes via frontal CBF. This pattern is consistent with the neurovascular unit serving as a potential interface between systemic vascular alterations and central nervous system dysfunction [34, 35]. Second, nominally significant indirect associations were observed between frontal CBF and behavioral outcomes via frontal GMV, consistent with the proposed relationship among cerebral hypoperfusion, structural neurodegeneration, and clinical impairment [36, 37]. However, none of these indirect associations remained significant after FDR correction, and six reverse mediation models also yielded nominally significant results. Consequently, the temporal direction and causality of these relationships cannot be determined from the present cross‐sectional data. These findings should therefore be considered hypothesis‐generating and require validation in larger longitudinal cohorts.

### 4.6 Strengths and Limitations

This study has several strengths. First, patients with probable bvFTD were diagnosed according to established consensus criteria and evaluated by a multidisciplinary team, while NC were matched for age, sex, and education. Second, the comprehensive clinical battery assessed cognitive, behavioral, and functional domains, allowing the clinical relevance of regional CBF to be examined across different manifestations of bvFTD. Third, ASL MRI, ^18^F‐FDG PET, structural MRI, and peripheral biomarkers were obtained within the same cohort, enabling direct cross‐modal comparisons. Finally, the analyses incorporated FDR correction and reverse mediation analyses, improving the transparency and robustness of the statistical evaluation.

Several limitations should be acknowledged. First, the cross‐sectional design precludes conclusions regarding temporal ordering or causality. Second, none of the indirect associations remained significant after FDR correction, and several reverse mediation models were nominally significant; therefore, the mediation findings are exploratory. Third, the modest sample size limited statistical power. Fourth, the inclusion of only patients with bvFTD and the absence of genetic or neuropathological classification restrict the generalizability of the findings. Finally, residual confounding by disease duration, genetic background, vascular risk factors, medication use, mood symptoms, or subtle pre‐existing regional abnormalities cannot be excluded. ASL measurements may also be affected by relatively low signal‐to‐noise ratio and arterial transit‐time effects.

### 4.7 Future Directions

Larger multicenter longitudinal studies are needed to confirm these findings and evaluate the diagnostic and prognostic value of ASL‐derived CBF. Head‐to‐head comparisons with ^18^F‐FDG PET should assess their relative and complementary clinical utility rather than assume equivalence or substitution. Future studies should include different FTD subtypes, genetic and neuropathological classifications, and more comprehensive vascular covariates. Paired plasma and CSF measurements may also clarify the biological origins of the peripheral biomarker findings. These approaches will help determine whether the observed neurovascular associations have clinical or therapeutic relevance.

## 5 Conclusion

In conclusion, bvFTD is characterized by frontotemporal hypoperfusion, with frontal CBF closely associated with cognitive, behavioral, and functional impairment. The regional concordance of CBF with GMV and glucose metabolism supports the potential role of ASL‐derived CBF as an accessible, non‐invasive, and radiation‐free complementary neuroimaging biomarker. The observed associations between peripheral vascular and synaptic biomarkers and frontal CBF, together with the exploratory mediation findings, suggest a potential neurovascular pathway linking peripheral alterations, cerebral perfusion, structural neurodegeneration, and clinical manifestations. Longitudinal studies are needed to establish the temporal direction and clinical significance of these relationships.

## Author Contributions

Liyong Wu and Min Chu designed and conceptualized the study. Jiahui Hou, Yufei Chen, Qianqian He, Yuxuan Xu, and Hua Lu acquired clinical and neuroimaging data. Jiahui Hou and Min Chu analyzed the images and performed the statistical analysis. Jiahui Hou and Liyong Wu drafted and revised the manuscript. All authors read and approved the final manuscript.

## Funding

This work was supported by grants from the Beijing New‐star Plan of Science and Technology Cross‐cooperation Project (20220484175), the National Natural Science Foundation of China (82401665), and Beijing Hospital Management Center “Peak” Talent Training Program (DFL20240805).

## Ethics Statement

This study was conducted following the Declaration of Helsinki. The clinical protocols were approved by the ethics committee and local institutional review board of Xuanwu Hospital, Capital Medical University, China (approval number: 2020026).

## Consent

The study was conducted following relevant guidelines and regulations for the use of human subjects in research. Written informed consent was obtained from all participants or their guardians before the start of the study.

## Conflicts of Interest

The authors declare no conflicts of interest.

## Supporting information


**File S1:** Receiver operating characteristic curves for the discriminative accuracy of frontotemporal CBF in differentiating bvFTD from normal control.
**File S2:** Associations between regional CBF and clinical scales in bvFTD patients.
**File S3:** Associations between regional CBF and peripheral biomarkers in bvFTD patients.
**File S4:** Mediation analyses between peripheral biomarkers and frontal CBF with clinical outcomes.
**File S5:** Mediation analyses between frontal CBF and frontal GM volume with clinical outcomes.
**File S6:** Reverse mediation analyses.

## Data Availability

The data that support the findings of this study are available from the corresponding author upon reasonable request.
