# Supplementary material for: Frontal Cerebral Blood Flow Deficits in Behavioral Variant Frontotemporal Dementia: Associations With Clinical Symptoms, Peripheral Vascular Biomarkers, and Neuroimaging Measures
Source: CNS Neurosci Ther. 2026 Nov 1;32(11):e71235. doi: 10.1002/cns.71235 (PMC13725197; doi:10.1002/cns.71235)
Supplement: Supplementary file 1 [file CNS-32-e71235-s001.docx]

**Contents**

Additional file 1. Receiver operating characteristic curves for the discriminative accuracy of frontotemporal CBF in differentiating bvFTD from normal control.........................................................2

Additional file 2. Associations Between Regional CBF and Clinical Scales in bvFTD Patients..............3

Additional file 3. Associations Between Regional CBF and Peripheral Biomarkers in bvFTD Patients..4

Additional file 4. Mediation analyses between peripheral biomarkers and frontal CBF with clinical outcomes ...…............................................................................................................................................5

Additional file 5. Mediation analyses between frontal CBF and frontal GM volume with clinical outcomes....................................................................................................................................................6

Additional file 6. Reverse mediation analyses...........................................................................................7

**Additional file 1. Receiver operating characteristic curves for the discriminative accuracy of frontotemporal CBF in differentiating bvFTD from normal controls.**

Abbreviation: ROC, Receiver operating characteristic; CBF, cerebral blood flow.

**Additional file 2. Associations Between Regional CBF and Clinical Scales in bvFTD Patients**

| **CBF** | **Clinical scales** | **β** | **P-value** | **FDR-P** |
| --- | --- | --- | --- | --- |
| Frontal_CBF | Memory | -0.743 | **<0.001** | **<0.001** |
|  | Orientation | -0.665 | **<0.001** | **0.001** |
|  | Judgement | -0.757 | **<0.001** | **<0.001** |
|  | SocialAffair | -0.707 | **<0.001** | **<0.001** |
|  | Hobby | -0.745 | **<0.001** | **<0.001** |
|  | Selfcare | -0.476 | **0.006** | **0.019** |
|  | Behavior | -0.410 | **0.021** | 0.052 |
|  | Language | -0.274 | 0.149 | 0.286 |
|  | FTLD-CDR | -0.883 | **<0.001** | **<0.001** |
|  | MMSE | 0.794 | **<0.001** | **<0.001** |
|  | MoCA | 0.790 | **<0.001** | **<0.001** |
|  | ADL | -0.609 | **<0.001** | **0.001** |
|  | NPI-Q | -0.351 | **0.050** | 0.104 |
|  | FBI-apathy | -0.402 | **0.029** | 0.069 |
|  | FBI-disinhibition | -0.519 | **0.002** | **0.008** |
|  | FBI-total | -0.488 | **0.006** | **0.018** |
| Temporal_CBF | Memory | 0.063 | 0.725 | 0.810 |
|  | Orientation | 0.133 | 0.475 | 0.621 |
|  | Judgement | 0.112 | 0.548 | 0.657 |
|  | SocialAffair | 0.142 | 0.425 | 0.611 |
|  | Hobby | 0.073 | 0.691 | 0.795 |
|  | Selfcare | 0.140 | 0.432 | 0.611 |
|  | Behavior | 0.243 | 0.169 | 0.311 |
|  | Language | -0.119 | 0.522 | 0.657 |
|  | FTLD-CDR | 0.131 | 0.479 | 0.621 |
|  | MMSE | -0.072 | 0.696 | 0.795 |
|  | MoCA | -0.032 | 0.862 | 0.919 |
|  | ADL | 0.190 | 0.287 | 0.476 |
|  | NPI-Q | 0.109 | 0.541 | 0.657 |
|  | FBI-apathy | 0.238 | 0.192 | 0.341 |
|  | FBI-disinhibition | 0.149 | 0.397 | 0.596 |
|  | FBI-total | 0.222 | 0.217 | 0.373 |

Abbreviation: CBF, cerebral blood flow; MMSE, Mini-Mental State Examination; MoCA, Montreal Cognitive Assessment; FTLD-CDR, Frontotemporal Lobar Degeneration Clinical Dementia Rating; ADL, Activities of Daily Living; NPI-Q, Neuropsychiatric Inventory Questionnaire; FBI, Frontal Behavioral Inventory.

**Additional file 3. Associations Between Regional CBF and Peripheral Biomarkers in bvFTD Patients**

| **CBF** | **Peripheral biomarker** | **β** | **P-value** | **FDR-P** |
| --- | --- | --- | --- | --- |
| Frontal_CBF | NPTXR | -0.345 | **0.046** | 0.100 |
|  | NPTX2 | -0.415 | **0.016** | **0.043** |
|  | VEGFA | -0.456 | **0.012** | **0.033** |
|  | PDGF-BB | -0.558 | **0.001** | **0.006** |
| Temporal_CBF | NPTXR | 0.022 | 0.901 | 0.940 |
|  | NPTX2 | 0.056 | 0.749 | 0.817 |
|  | VEGFA | 0.001 | 0.994 | 0.994 |
|  | PDGF-BB | -0.136 | 0.460 | 0.621 |

Abbreviation: CBF, cerebral blood flow; NPTXR, neuronal pentraxin receptor; NPTX2, neuronal pentraxin 2; VEGFA, vascular endothelial growth factor A; PDGF-BB, platelet-derived growth factor BB.

**Additional file 4. Mediation analyses between peripheral biomarkers and frontal CBF with clinical outcomes**

| **X_Variable** | **Y_Variable** | **Mediator** | **ACME_β** | **ACME_p** | **ADE_β** | **ADE_p** | **Total Effect_β** | **Total Effect_p** | **Prop_Mediated** |
| --- | --- | --- | --- | --- | --- | --- | --- | --- | --- |
| PDGFBB | Orientation | Frontal_CBF | 0.000 | 0.004 | 0.000 | 0.412 | 0.000 | 0.008 | 0.682 |
| PDGFBB | Judgement | Frontal_CBF | 0.000 | 0.002 | 0.000 | 0.196 | 0.000 | 0.000 | 0.620 |
| PDGFBB | Selfcare | Frontal_CBF | 0.000 | 0.048 | 0.000 | 0.484 | 0.000 | 0.048 | 0.593 |
| PDGFBB | FTLD-CDR | Frontal_CBF | 0.002 | 0.002 | 0.000 | 0.616 | 0.002 | 0.000 | 0.877 |
| PDGFBB | MMSE | Frontal_CBF | -0.003 | 0.002 | -0.001 | 0.226 | -0.004 | 0.002 | 0.682 |
| PDGFBB | ADL | Frontal_CBF | 0.004 | 0.004 | 0.001 | 0.578 | 0.006 | 0.018 | 0.736 |
| VEGFA | Judgement | Frontal_CBF | 0.004 | 0.006 | 0.001 | 0.600 | 0.005 | 0.022 | 0.791 |
| VEGFA | Selfcare | Frontal_CBF | 0.002 | 0.048 | 0.002 | 0.204 | 0.004 | 0.014 | 0.413 |
| VEGFA | FTLD-CDR | Frontal_CBF | 0.023 | 0.006 | 0.005 | 0.518 | 0.028 | 0.002 | 0.826 |
| VEGFA | MMSE | Frontal_CBF | -0.032 | 0.006 | -0.011 | 0.438 | -0.042 | 0.020 | 0.739 |
| VEGFA | ADL | Frontal_CBF | 0.045 | 0.008 | 0.041 | 0.196 | 0.086 | 0.008 | 0.518 |

Abbreviation: CBF, cerebral blood flow; MMSE, Mini-Mental State Examination; FTLD-CDR, Frontotemporal Lobar Degeneration Clinical Dementia Rating; ADL, Activities of Daily Living.VEGFA, vascular endothelial growth factor A; PDGF-BB, platelet-derived growth factor BB; ADE, Average Direct Effect; ACME, Average Causal Mediation Effect.

**Additional file 5. Mediation analyses between frontal CBF and frontal GM volume with clinical outcomes.**

| **X_Variable** | **Y_Variable** | **Mediator** | **ACME_β** | **ACME_p** | **ADE_β** | **ADE_p** | **Total Effect_β** | **Total Effect_p** | **Prop_Mediated** |
| --- | --- | --- | --- | --- | --- | --- | --- | --- | --- |
| FRONTAL_CBF | Behavior | Frontal_GMV | -0.047 | 0.002 | -0.020 | 0.502 | -0.067 | 0.016 | 0.701 |
| FRONTAL_CBF | FBI-apathy | Frontal_GMV | -0.395 | 0.028 | -0.237 | 0.418 | -0.632 | 0.022 | 0.612 |
| FRONTAL_CBF | FBItotal | Frontal_GMV | -0.512 | 0.038 | -0.633 | 0.140 | -1.145 | 0.006 | 0.445 |

Abbreviation: CBF, cerebral blood flow; GMV, gray matter volume; ADE, Average Direct Effect; ACME, Average Causal Mediation Effect.

**Additional file 6. Reverse mediation analyses**

| **X_Variable** | **Y_Variable** | **Mediator** | **ACME_β** | **ACME_p** | **ADE_β** | **ADE_p** | **Total Effect_β** | **Total Effect_p** | **Prop_Mediated** |
| --- | --- | --- | --- | --- | --- | --- | --- | --- | --- |
| ADL | PDGF-BB | Frontal_CBF | 0.289 | 0.012 | 0.122 | 0.543 | 0.408 | 0.023 | 0.684 |
| Self-care | PDGF-BB | Frontal_CBF | 0.235 | 0.010 | 0.136 | 0.459 | 0.368 | 0.044 | 0.604 |
| Orientation | PDGF-BB | Frontal_CBF | 0.267 | 0.031 | 0.170 | 0.393 | 0.438 | 0.011 | 0.595 |
| Behavior | Frontal_CBF | Frontal_GMV | −0.275 | 0.016 | −0.132 | 0.490 | −0.405 | 0.021 | 0.667 |
| FBI_apathy | Frontal_CBF | Frontal_GMV | −0.236 | 0.010 | −0.138 | 0.429 | −0.373 | 0.029 | 0.621 |
| FBI_total | Frontal_CBF | Frontal_GMV | −0.214 | 0.018 | −0.255 | 0.150 | −0.467 | 0.006 | 0.449 |

Abbreviations: CBF, cerebral blood flow; GMV, gray matter volume; ADE, Average Direct Effect; ACME, Average Causal Mediation Effect.
